# Investigation of Shear-Induced Deformation of Reinforcing Textiles by Optical Measurement Devices

**DOI:** 10.3390/ma12071029

**Published:** 2019-03-28

**Authors:** Stefan Rothe, Ellen Wendt, Sybille Krzywinski, Marianna Halász, Peter Bakonyi, Peter Tamás, Attila Bojtos

**Affiliations:** 1 Institute of Textile Machinery and High Performance Material Technology (ITM), TU Dresden, 01062 Dresden, Germany; ellen.wendt@tu-dresden.de (E.W.); sybille.krzywinski@tu-dresden.de (S.K.); 2 Department of Polymer Engineering, Budapest University of Technology and Economics, 1111 Budapest, Hungary; halaszm@pt.bme.hu (M.H.); bakonyi@pt.bme.hu (P.B.); 3 Department of Mechatronics, Optics and Engineering Informatics, Budapest University of Technology and Economics, 1111 Budapest, Hungary; tamas@mogi.bme.hu (P.T.); bojtos@mogi.bme.hu (A.B.)

**Keywords:** textiles, composite preforming, mechanical properties, shear behavior, surface analysis, picture frame test, kinematic draping simulation

## Abstract

When fiber-reinforced plastic (FRP) components are designed, it is very important to ensure that textiles are formed into complex 3D geometries without folds, and that the reinforcing structure is oriented appropriately. Most research in this context is focused on finite element (FE) forming simulations and the required characterization of textile reinforcements. However, the early stage of the design of FRPs, where kinematic draping simulations are used, is barely considered. In particular, the need for a critical shear angle for the execution and evaluation of kinematic draping simulations is often neglected. This paper presents an extended picture frame test stand with an optical device recording shear-induced deformations with the help of a laser line emitter. Associated hardware and software for detecting and quantifying the fold formation during a picture frame test were developed. With the additional recorded information, a material-specific critical shear angle can be determined, material behaviors can be compared, and FE-based simulation methods can be evaluated. This innovative test stand and the associated software tools will help engineers to decide on suitable materials and improve transparency in the early stages of the design process.

## 1. Introduction

The importance of energy and resource efficiency is constantly growing, consequently, the significance of innovative and interdisciplinary lightweight technologies rises as well. This applies, in particular, to passenger and freight transport, and mechanical and plant engineering [1]. For this reason, high-strength fiber-reinforced plastics (FRPs) are increasingly used, in addition to traditional construction materials, such as aluminum or steel. Due to their fibrous structure, high formability and adjustable directional properties, textile reinforcements (e.g., woven, knitted, braided or laid fabrics) are commonly used in the manufacturing of FRPs [2].

Such FRPs are lightweight, but expensive and often require a high effort of manual labor. Nevertheless, a double-digit growth of carbon fiber-reinforced plastics is expected by 2020 [3]. Therefore, it is crucial to significantly reduce costs by shortening product development cycles and making manufacturing resource-efficient. Computer-aided methods can greatly help with this as they are particularly suitable for complex and even double-curved FRP products. Especially in the early stages of designing an FRP, when various models are considered, costs can be avoided with kinematic draping simulations. In contrast to FE-based simulations, which are suitable for the subsequent planning of automated processes, kinematic draping simulations are faster, easier to handle and less sophisticated with regard to material characterization. Nevertheless, the manufacturing process is taken into account by predefined handling methods. Two important aspects in evaluating kinematic draping results and FE-based forming simulations are the fold-free forming of reinforcing textiles and a load-adapted orientation of reinforcing fibers. In particular, folds occurring during manufacturing must be strictly avoided as they lead to a significant reduction in strength or impaired damage behavior of the final FRP product [4,5].

While numerous investigations focus on FE-based simulation methods and the related characterization of materials [6,7,8,9,10], kinematic draping simulations and their requirements are investigated sparsely. However, only one parameter that limits the formability is needed for the assessment of a kinematic draping simulation. In available software solutions and in the industry, this material-specific parameter is often named “critical shear angle”, which is not the same as the “locking angle” as used in the FE-based simulations literature. The “critical shear angle” can be determined by shear behavior testing, although there is no standardized method yet. The investigations and the developed test stand presented in this paper aim to determine the critical shear angle reproducibly and comprehensibly for reliable kinematic draping simulations, and to give an assessment in the product development cycle.

### 1.1. Determination of Shear Deformation Behavior

Besides tensile and flexural behavior, shear behavior is essential for assessing the overall deformation behavior of reinforcing textiles [11,12,13]. While the former two are evaluated according to standardized test methods [1], no standard for the determination of shear behavior exists yet, in spite of numerous investigations regarding 2D and 3D characterization [14]. This obviously shows the difficulty of determining textile shear behavior. In developing a shear test method, three assumptions are made for textiles based on less extensible fibers, e.g., glass or carbon: The fibers are inextensible, no slippage occurs between fibers and the flexural stiffness of fibers is negligible [15]. These assumptions are the same as the assumptions in kinematic draping simulations [16,17,18]. Therefore, shear behavior tests with these assumptions are suitable for determining the “critical shear angle”. This angle is defined here as the shear angle at which a specific fold height occurs during shear testing.

The shear process of textiles is divided into two main shear principles: pure shear and simple shear [19]. For simple shear, the distance between the two clamping lines remains constant during the shear process. This leads to an elongation of the unclamped sample edges. It not only changes the angle between the yarns, but also causes tensile strain due to yarn torsion at the crossing points. In contrast, pure shear only changes the angle between the yarns without yarn elongation, because the distance between the clamping lines does not remain constant. Based on these assumptions, shear processes inducing both pure and constant shear on textiles are preferred, e.g., the bias extension test or picture frame test [12].

#### 1.1.1. Bias Extension Test

**Figure 1:**
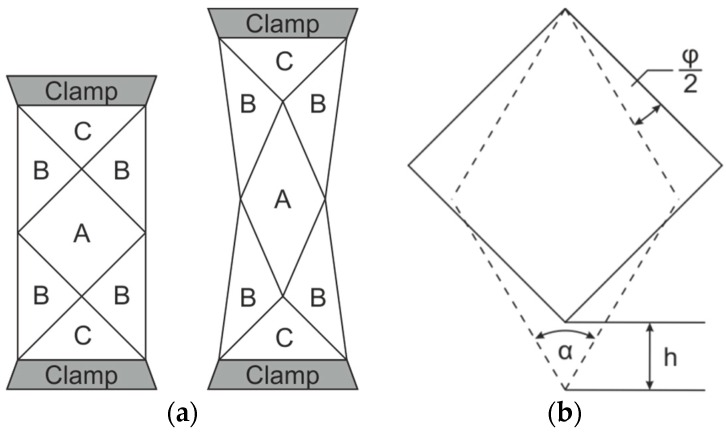
Principle of shear deformation in the bias extension test (**a**) and in the picture frame test (**b**) [1].

The bias extension test (BET) is widely used because of its simplicity. A 45° oriented textile sample is fixed into a tensile testing device and loaded with tension [20,21,22]. Additionally, the BET can be performed as a biaxial tensile test with a modified sample geometry and clamping in the second direction [23]. In a standard clamping arrangement, there are different shear zones, Figure 1a. In zone A, pure and constant shear is induced, due to the free edges of the relevant yarns. In zone B, only half the shear of zone A is induced due to the partly clamped yarns. Finally, in zone C, no shear occurs due to the completely clamped yarns. During testing, the force/displacement diagram is recorded. However, the measured force reflects the whole effort of sample shearing. As this effort depends on the sample size, a normalized shear force was introduced [12]. Calculating the normalized shear force requires a comprehensive knowledge of engineering mechanics.

As mentioned above, the BET is widely used and well-investigated. Most of the research only considers woven fabrics as to their “pin-joint” arrangement. In several studies, a material-specific locking angle was determined, describing the shear angle when “locking” occurs. The locking phenomenon is observed when warp and weft yarns cannot rotate further without inducing non-negligible in-plane compressions on their neighboring yarns. As it is not possible to measure the moment of locking directly, it is derived from the shear force/shear angle diagram or by additional optical measurements. Theoretically, the shear force/shear angle diagram shows a steep rise at the moment of locking, so it can be derived quite easily. However, it was shown in [24] that folding was observed before locking. This additionally supports the distinction in the terminology used. In [25], it is shown that folding depends on textile properties, whole strain and stress distribution and on boundary conditions. Folding is mentioned as a multiscale problem. Thus, the determination of the locking angle does not seem to be a reliable criterion for deriving the critical shear angle needed for kinematic draping simulations.

In addition, there are a few studies also investigating multi- and uni-directional non-crimp textiles (NCF and UD-NCF, respectively) [26,27,28,29]. For NCFs, slippage was observed starting at a shear angle of 40° [27]. This is a major disadvantage of BET and leads to a minor deviation between the mathematically calculated shear angle and the optically measured shear angle. Consequently, the applicability of BET is questionable for NCFs, because of the missing pin joint arrangement and the spuriously made assumption regarding slippage. Nevertheless, such effects are also encountered in the practice of manufacturing FRP products and need to be considered.

Optical measurements of the out-of-plane deformation behavior during BET are rare. In [23], a multistep biaxial bias extension test was performed to measure the force required to unfold an already sheared woven fabric sample. At each step, the resulting deformation was 3D scanned. Therefore, the sample and its surroundings needed to be additionally prepared, as compared to the standard bias extension test. The scans were edited to achieve smooth evaluable surfaces and the dimensions of the fold were derived. This procedure was not aimed at determining a critical shear angle as needed for kinematic draping simulations, but it can be used for the comparison of materials. It is not a continuous measurement of folding, i.e., the beginning of folding cannot be determined. In addition, sample preparation, the test procedure, data preparation, and evaluation are time consuming and cost intensive.

#### 1.1.2. Picture Frame Test

In the picture frame test (PFT) [12,21,29,30], pure and constant shear is achieved by fixing the edges of a square sample (initial state α = 90°) in a hinged frame. Tensile loading at two of its opposite corners causes the sample to deform into a rhomboid shape depending on transversal displacement h (Figure 1b). In contrast to BET, the induced shear is uniform. The force and displacement are recorded analogously to BET. The sample shear angle *φ* can be calculated with the use of the transversal displacement h and the picture frame edge length a, as in Equation (1):

(1) $$\varphi =90{}^{\circ}-2\times \cos^{-1}\left(\frac{1}{\sqrt{2}}+\frac{h}{2\times a}\right)$$

The shear force *F_sh_* can be calculated directly for standard hinged picture frames depending on the shear angle, with Equations (2) and (3) [30,31]:

(2) $$F_{sh}=\frac{F}{2\times \cos\alpha }$$

(3) α=90°−φ

A group of academic and industrial researchers conducting suitable picture frame test methods as well as BET methods was set up for benchmarking. Five picture frame devices were investigated and compared [12]. All presented picture frames were based on rigid clamps, but the dimensions differed and consequently lead to different sample sizes. Three normalization methods were compared. Finally, a normalization method with an energy approach considering both the side length of the fabric and of the picture frame [30] was recommended [12]. Besides different dimensions, two picture frames use a lever mechanism, for which the amplification needs to be considered in the calculation of shear force. A main disadvantage of all presented picture frames were the induced in-plane tensions due to rigid clamping as they significantly influence the beginning of folding [25,32]. Additionally, the frame itself and each sample require elaborate preparation. In particular, precise alignment of the sample while being clamped on the frame is essential, or else additional tensions are generated, which also cause folding to begin earlier.

The applicability of the PFT has been investigated for several textiles, mainly for woven fabrics, but also for UD-NCF [26] and pre-consolidated textiles [33]. Furthermore, BET and PFT have been compared extensively [20,21,22,34]. Cao et al. [12] investigated balanced twill and plain woven fabrics, as well as unbalanced twill woven fabrics with both tests. It was shown that the PFT is feasible for balanced woven fabrics, but not for unbalanced woven fabrics. The obtained shear force/shear angle diagrams were comparable to the results of BET after normalization. Additionally, different preparation methods have also been investigated, leading to the conclusion that mechanical conditioning (e.g., shearing the sample a few times by hand) increases reproducibility. However, it was considered that such a procedure is not feasible in industry. Another conclusion was that it is necessary to remove transverse yarns from the clamping arms to prevent the initiation of folding in these zones, due to the rigid clamps [35]. A comparison of woven fabrics and UD-NCFs with adapted clamping revealed that shear tests are not suitable for UD-NCFs, as shearing is not an intrinsic behavior either in PFT or BET [26]. For pre-consolidated textiles, alternative sample preparation was needed to allow PFT, otherwise clamping would have prevented shearing due to the consolidated state of the material. The authors mentioned that the locking angle cannot be determined by PFT for pre-consolidated textiles. Furthermore, the definition of locking angle needs to be reconsidered. In [36], the theoretical determination of the locking angle based on textile parameters for one specific woven fabric is described and experimentally investigated by PFT and BET. Due to the test procedure of PFT, where intra-plane yarn shear was induced, it was found that locking theoretically occurs at higher angles than in BET. This was confirmed with the experiments for PFT and BET. Additionally, the authors assumed that folding would appear after locking. Even if this was confirmed by their experiments, other investigations showed diverging tendencies, i.e., this observation cannot be generalized.

Other investigations of the last decade have mainly focused on FE-based simulation methods. Therefore, the relationship between tension and shear behavior was examined extensively to improve simulation models and results of forming simulations. In addition, there are several studies dealing with the purposeful generation of pretensions in shear testing and in manufacturing to prevent folding or delay the onset of folding. These studies are necessary for FE-based simulations and contribute to a better understanding of textile deformation behavior in general. However, they are less essential for kinematic draping simulations as they do not determine the critical shear angle or consider the global phenomenon of folding.

**Figure 2:**
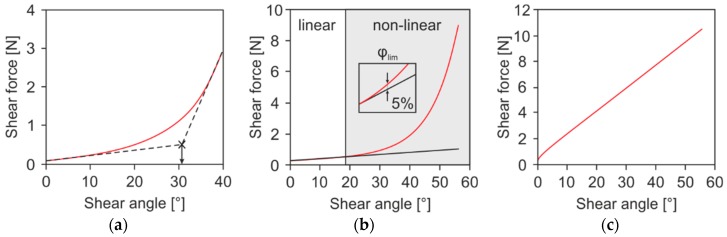
Evaluation of a shear force/shear angle diagram. Determination of the critical shear angle (**a**) according to [37] and (**b**), according to [38,39], (**c**) shear force/shear angle diagram, biaxial non-crimp fabric ±45°, sewing thread (ST) 0°.

Nevertheless, the achieved shear force/shear angle data can be used for determining the critical shear angle by further data analysis. Souter [37] introduced a method to identify the critical shear angle by intersecting two fitting linear regressions in the first and last five degrees of the shear force/shear angle data (Figure 2a). Another method is described in [38,39], where a linear regression is fitted in the shearing phase at the very beginning of the shear force/shear angle data. Once the measured data exceed a deviation of 5% or more as compared to linear regression, the critical shear angle is obtained (Figure 2b).

However, both methods are questionable for several reasons. First of all, these shear force/shear angle diagrams correspond to ideal shear behavior. For realistic data sets, it is more challenging to define different linear zones (Figure 2c). Also, the subjective definition of linear zones leads to minor deviations. Additionally, in Souter’s method, the definition of the posterior linear zone is strongly dependent on the procedure parameter of maximum transverse distance, and consequently, influences the intersection point. As can be seen, the post-determination of critical shear angle from shear force/shear angle data is not reliable and prone to error.

Of course, this fact is known and has led to several approaches to determine the critical shear angle in a shear test. Non-destructive optical measurements are suitable for this purpose as the critical shear angle depends on folding. A widely used optical method is DIC (Digital Image Correlation), which can be used to capture local elongations. The distribution of elongations over the sheared samples is examined and evaluated in the matter to determine the critical shear angle. This procedure is very elaborate in regards to data processing. In [39], the distribution of elongation on a PFT sample was averaged at each transversal distance step. As folding occurred, the mean value of elongation rose significantly and was visible in the overall diagram. However, no information of the folding dimensions was obtained.

Another approach is to identify folding by means of a gray-scale image. However, subsequent image analysis is extremely time-consuming and the results are often inaccurate. Commercial 3D deformation detection systems are provided by GOM. However, this technique is quite costly and laborious in terms of sample preparation (PONTOS system) and the evaluation of the recorded data (ARAMIS system). Our own experience has shown that both solutions are prone to error as data points were missing during the progressive test process.

The lack of reproducibility and traceability as well as the discontinuity and high effort of existing solutions (for both PFT and BET) for determining the critical shear angle by means of fold dimensions were the main drivers for the developed test stand presented in this study. This initial situation motivated the joint research project of the Institute of Textile Machinery and High Performance Material Technology of TU Dresden, and the Department of Polymer Engineering of TU Budapest. The objective was to exploit the detection and quantification of folds during the picture frame test and, subsequently, to achieve a cost-efficient solution. Therefore, we investigated image processing methods in terms of suitability and added necessary equipment to the picture frame test stand.

## 2. Materials and Methods

**Figure 3:**
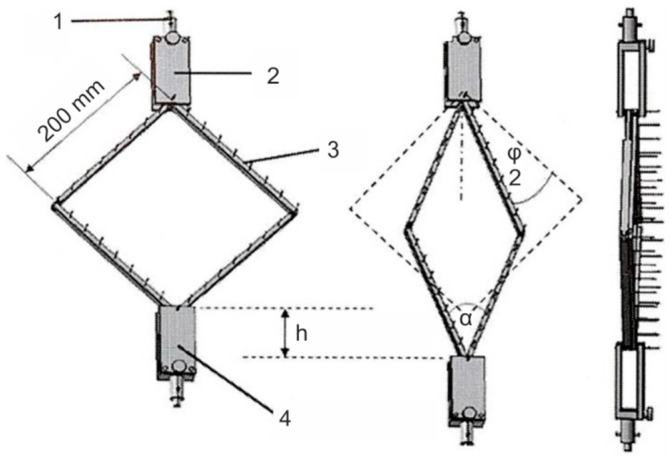
Modified picture frame (1: adapter; 2: upper frame holder; 3: needle bar; 4: lower frame holder).

The investigations were based on the picture frame shear test, described above in Section 1.1.2. A picture frame modified at the ITM was used [19]. In contrast to the commonly used picture frames, with a clamping mechanism holding the samples at their edges, the samples are held by needle bars. This allows the twisting of the reinforcing threads and the force to be transferred from the picture frame into the sample (Figure 3). This picture frame test stand was completed by an option for the continuous optical detection of shear deformation. During the picture frame test, fold deformation is most pronounced at the center of the sample; it is therefore sufficient to measure the central deflection of the sample. In this respect, our device is different from commercially available 3D deformation measuring systems offered by the company GOM or by a recent study [23], which includes the deformation of the entire surface.

The test conditions were as follows:

Test device:Monoaxial tensile test machine Z 2.5, ZWICK, Ulm, GermanyForce sensor:500 NSample size:300 mm × 300 mmPicture frame edge length:200 mm × 200 mmDeformation distance:60 mm (corresponds to a shear angle of *φ* ≈ 28°)Test speed:100 mm/minTest conditions:Standard climate (DIN EN ISO 139), *T* = 20 °C, rel. humidity = 65%Recorded data:Force *F* at increments of Δ*h* = 0.1 mm in transverse direction Displacement

**Figure 4:**
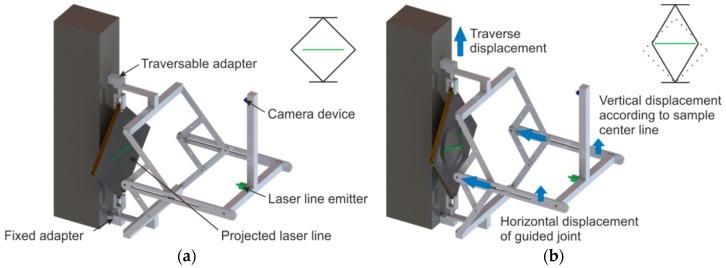
Extended picture frame test stand. Initial (**a**) and deflected (**b**) state.

**Figure 5:**
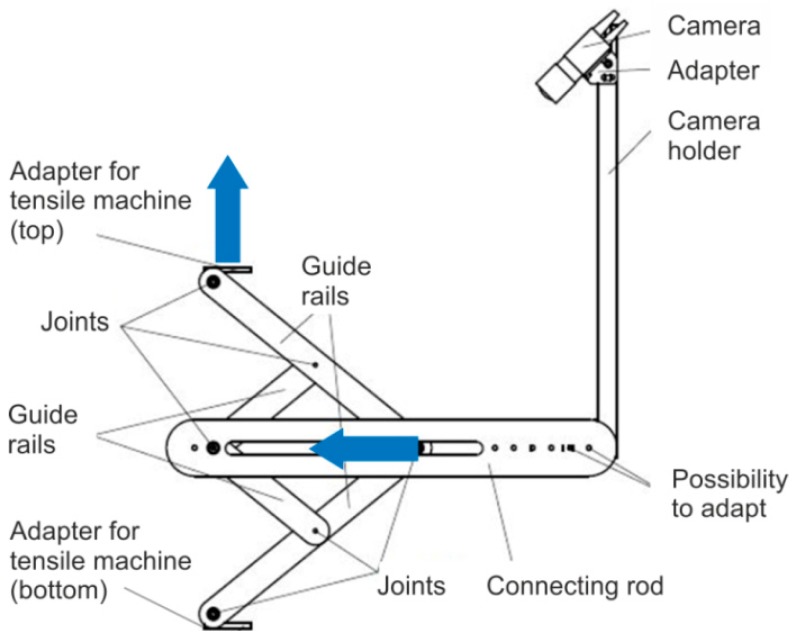
Setup of the extended picture frame test stand.

The extended test setup consists of a device including a laser line emitter, a camera and a computer with specific data processing software. The equipment is attached to the tensile testing machine in addition to the picture frame. The attachment is above the force sensor so that it does not influence the capture of the shear force. The laser line emitter, which is fixed at a defined distance, projects a horizontal beam orthogonally onto the sample surface (constant over 150 mm width). A Basler acA 14 µm camera (Basler AG, Ahrensburg, Germany) for the optical recording of the laser beam is placed at a defined angle and distance to the sample plane (Figure 4 and Figure 5). Due to its kinematic design with fixed and guided joints, the equipment ensures a constant position of the projected laser beam in the middle of the sample (Figure 4b). The distance of laser line emitter and camera remains constant over testing.

**Figure 6:**
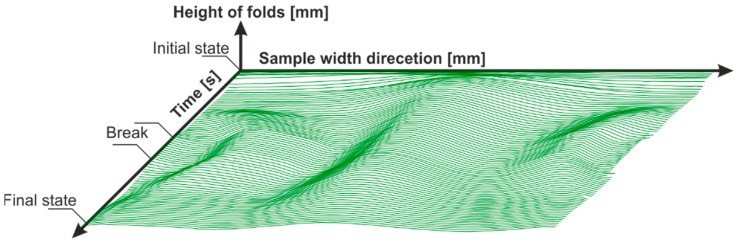
Measured height profile of the laser beam as a function of time (SDK software, Basler) over a constant width of 150 mm—carbon fiber (CF) biaxial non-crimp fabric ±45°, ST 0°; Hysteresis with 10 seconds break at maximum deflection.

During the shear test, the geometrical change of the laser beam caused by the shear deformation of the sample was continuously recorded with a Software Development Kit (SDK) from Basler AG. In the initial state, the distance between the left and right joints of the shear frame was 282.8 mm. With a shear deformation of 28° (corresponding to a deflection of 60 mm), the distance was 206 mm. The laser beam was captured over a constant width of approx. 150 mm during the picture frame test. Image processing software supported by OpenCV calculated the deflection curves of the laser beam for every single time step. After calibration (see Section 3.1) and interpolation, the geometric data of the deflection curves were determined as a function of time (Figure 6) and were available for further analysis.

**Figure 7:**
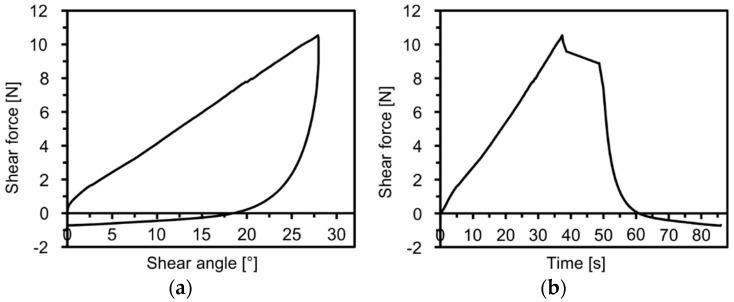
Shear force/shear angle diagram (**a**). Shear force/time diagram (**b**) for a CF-biaxial non-crimp fabric.

The error of the measurement system resulting from an incompletely level position of the sample is eliminated by comparing the geometry data of each time step with the first time step (initial state). The force/displacement diagram and the shear force/shear angle diagram are recorded simultaneously in accordance with the optical detection of the laser beam. This allows a distinct force and a corresponding shear angle to be related to the respective shear deformation for each time step (Figure 7).

### Device Calibration

It is necessary to calibrate the data recorded by the camera for accuracy. Data evaluation is based on the theory of 3D scanning (triangulation).

The laser beam projects a planar curve onto the material to be measured. Taking a picture of the projected curve on the material surface is a plane-to-plane perspective transformation as a bijection. A perspective transformation by homogenous coordinates is a linear transformation [40] projecting a quadrangle to a quadrangle. The transformation matrix has eight independent coordinates (*p*_0_, *p*_1_, …, *p*_7_), as shown in Equation (4):

(4) $$\underset{=}{P}=\left[\begin{array}{ccc} p_{0} & p_{1} & p_{2}\\ p_{3} & p_{4} & p_{5}\\ p_{6} & p_{7} & 1 \end{array}\right]$$

**Figure 8:**
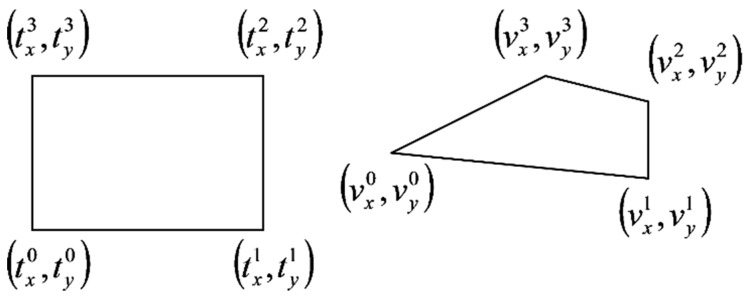
Planar perspective projection.

Corners of rectangular calibration equipment are appropriate to define the matrix coordinates (Figure 8).

The corners of the calibration equipment are $\left(t_{x}^{i},t_{y}^{i}\right)$, and the corners of its picture are $\left(v_{x}^{i},v_{y}^{i}\right)$ (i = 0, 1, 2, 3); based on this, the transformation is shown in Equation (5):

(5) $$\left[\begin{array}{c} v_{x}^{i}\\ v_{y}^{i}\\ 1 \end{array}\right]=\left[\begin{array}{ccc} p_{0} & p_{1} & p_{2}\\ p_{3} & p_{4} & p_{5}\\ p_{6} & p_{7} & 1 \end{array}\right]\cdot \left[\begin{array}{c} t_{x}^{i}\\ t_{y}^{i}\\ 1 \end{array}\right]$$

There are eight unknown coordinates and eight equations, Equation (6):

(6) $$\left[\begin{array}{c} v_{x}^{i}\\ v_{y}^{i}\\ 1 \end{array}\right]=\left[\begin{array}{ccc} p_{0} & p_{1} & p_{2}\\ p_{3} & p_{4} & p_{5}\\ p_{6} & p_{7} & 1 \end{array}\right]\cdot \left[\begin{array}{c} t_{x}^{i}\\ t_{y}^{i}\\ 1 \end{array}\right]\;i=0,1,2,3$$

Knowing the real positions of four points in the lighted plane (*t^i^*) allows the real coordinates of other points on the lighted plane to be computed.

**Figure 9:**
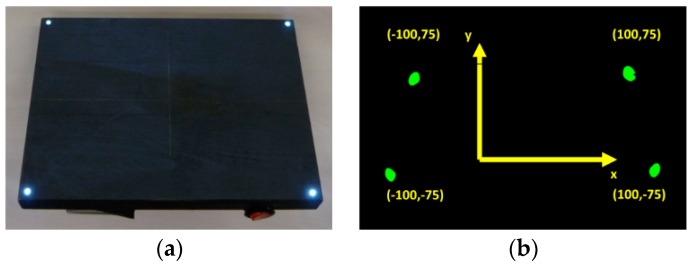
The calibration device (**a**) and its image during calibration (**b**) with its real dimensional coordinates.

The system is calibrated with a rectangular device with an LED in each corner (Figure 9a). During the calibration process, the rectangle is positioned in the laser lighted plane. This means that the device is aligned in the picture frame in such a way that it is positioned centrally and planarly in all planes, sagittal, transverse and frontal, with the frontal plane corresponding to the position of the shear specimen in the initial state. Figure 9b shows an image of the calibration tool. With the measured picture coordinates and the real coordinates of the LEDs, the eight coordinates of the homogenous transformation matrix can be calculated.

**Figure 10:**
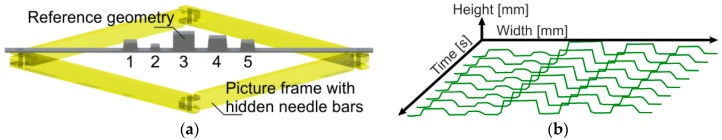
Reference geometry with defined peaks (1 to 5) adapted to the picture frame (**a**) and its measured height profile (**b**).

In order to validate the hardware and software for recording and evaluating the shear deformation that occurs during the picture frame test, a reference geometry with a defined height profile (Figure 10a) was designed and manufactured. The aim of validation is to ensure that the height profile is measured correctly. Since shear deformation is only measured centrally, based on the deflection of the projected laser beam, the dimensions of the reference geometry do not need to correspond to the sample geometry, only allow the projection of the laser beam. For this purpose, the reference geometry was positioned on the lateral pivot point of the square picture frame and the laser beam is projected. The optical measurement of this height profile and its comparison to nominal dimensions is made stationary state and requires all measuring system components to be properly aligned and a validated calibration.

For experimental investigations, different reinforcing textiles which are widely used, e.g., in automotive and wind power plant engineering, were selected, Table 1. They vary in terms of fabric construction and/or fiber material, e.g., CF, glass fiber (GF), polyamide (PA) or polyester (PES), thus representing the group of commonly investigated materials.

## 3. Results

To determine the accuracy of the extended picture frame test stand, the reference geometry was measured after calibrating. The deviations between measured and nominal height were mostly less than 5%, Table 2. Only at peak 4 (nominal height = 15 mm) the deviation was marginally higher than 5%. This could be caused by the averaging over relevant data points of this plateau of peak. Defining those relevant data points is quite challenging, because from the gained data, it is not clear whether a data point belongs to the plateau of the peak or to the very beginning of the inclined flank. In the visualization of data set points the inclined flanks can be seen (Figure 10b). Minor deviations in horizontal direction result from the limited number of data set points, which are only linearly connected for visualization. This effect is less relevant for plain and sheared textiles compared to the smooth surfaces without any hard transitions.

**Figure 11:**
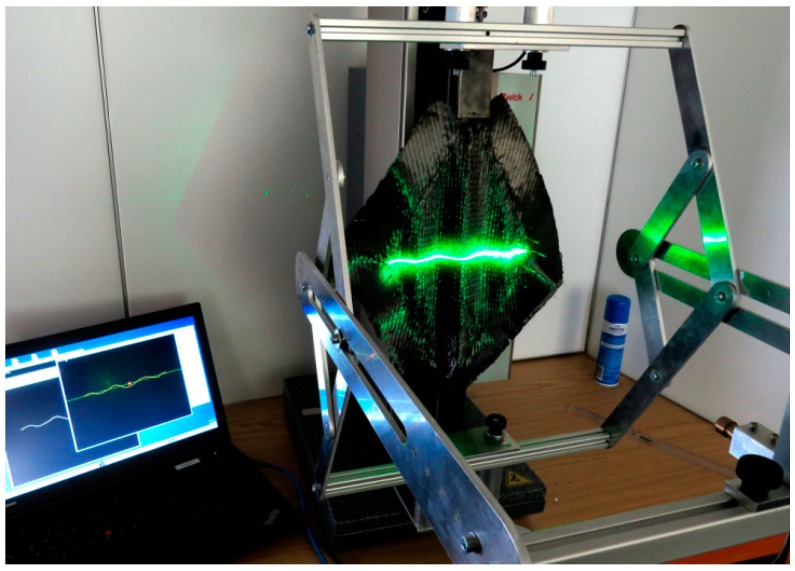
Extended picture frame test stand with CF biaxial non-crimp ± 45°, ST 0° at maximum deflection.

**Figure 12:**
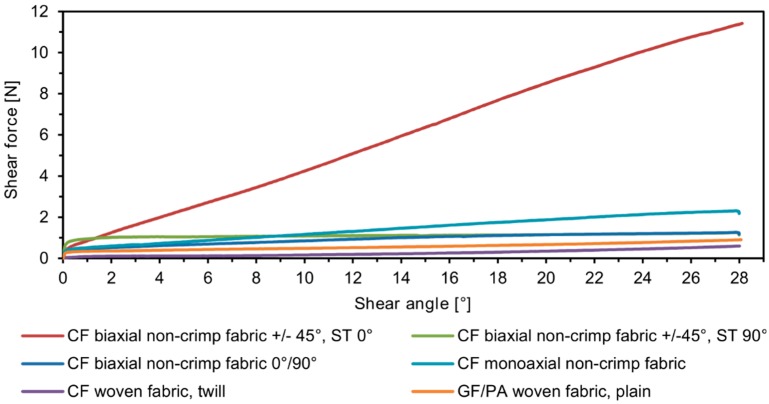
Shear force/shear angle diagram of investigated materials (average curve from nine samples for each material).

**Figure 13:**
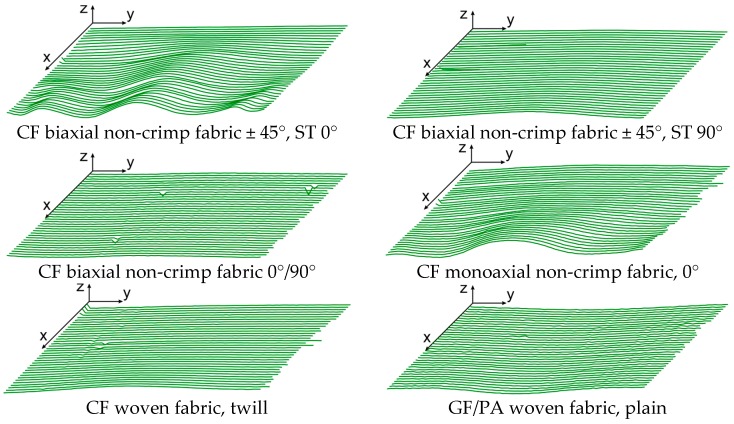
Measured height profiles of the laser beam as a function of time (one sample per material is shown); x-axis: time; y-axis: width; z-axis: height (also see Figure 6).

The selected materials were investigated (Figure 11), the force/displacement diagram was obtained and the respective deflection of the laser beam was measured optically over time. Due to its fabric construction, the ± 45° CF biaxial non-crimp fabric was tested in two directions to investigate the influence of the sewing thread (in test direction/90° relative to test direction) on shear behavior. In order to avoid errors in the optical detection due to reflections of the tested reinforcing textiles (glass/carbon), the samples were treated with a digitizing spray in the area of the laser beam. This had no effect on the shear result. Subsequently, the shear force, the shear angle (Figure 12), and the geometrical data of shear deformation in the area of the laser beam (Figure 13) were calculated. 

**Figure 14:**
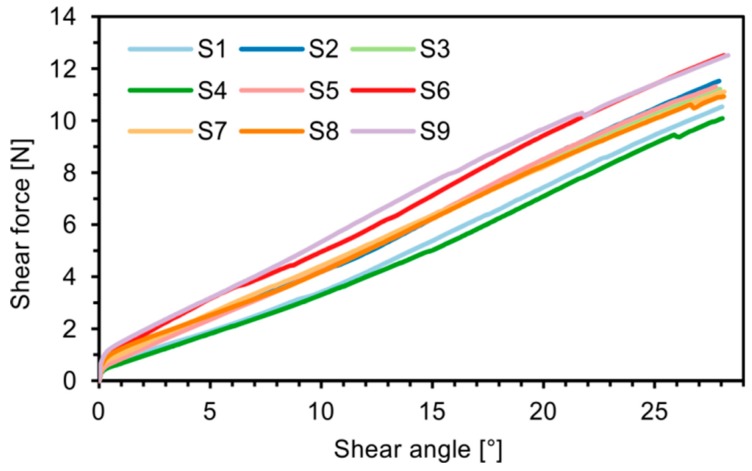
Shear force/shear angle diagram—CF biaxial non-crimp fabric ± 45°, ST 0°.

**Figure 15:**
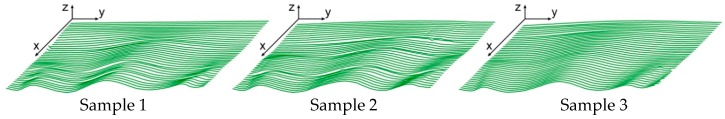

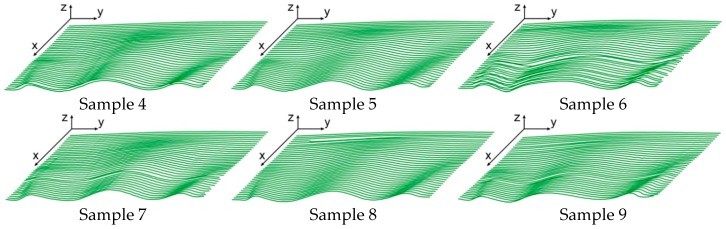
Measured height profiles of the laser beam as a function of time—CF biaxial non-crimp fabric ± 45°, ST 0°; x-axis: time; y-axis: width; z-axis: height (also see Figure 6)

To evaluate the reproducibility of the results, nine samples were tested for each material and material direction. Figure 14 and Figure 15 show the shear force/shear angle curves as well as the recorded surface deformations for the ± 45° biaxial non-crimp fabric with the sewing thread in the tensile direction. The deviations of about 20% in the shear force/shear angle curve result from the properties of the semi-finished product caused by fabric construction. For non-crimp fabric the yarn systems are not crossed but held by a sewing thread to ensure good drapeability. The missing crossing lead to a nearly perfectly stretched out situation of the reinforcing fiber. Thus, high mechanical characteristics in the reinforced directions (e.g., mono-, bi- tri- or quadriaxial) can be realized in FRP products. But its low resistance to displacement, restricts its handleability, making it almost impossible to achieve exactly reproducible sample preparation in the shear test.

The recorded surface deformations show generally similar results (Figure 15). Minor differences in the characteristics are the result of the above-mentioned issues in sample preparation. Moreover, it is obvious that similar shear force/shear angle curves do not lead to similar deformation behavior. To determine the exact beginning of fold formation and the geometric fold characteristics under shear stress, further analysis of the measured surface deformation is needed.

### 3.1. Additional Options for the Analysis of Optically Measured Shear Deformation

**Figure 16:**
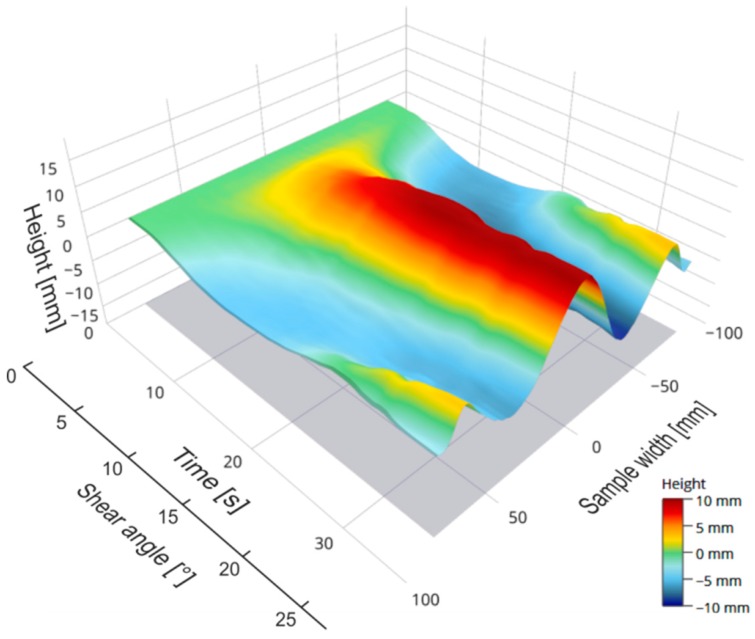
Deflection of the laser beam over time of CF biaxial non-crimp fabric ± 45°, ST 0°.

**Figure 17:**
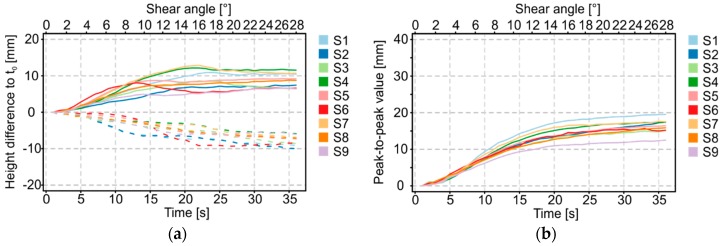
Amplitudes of maxima and minima (**a**). Peak-to-peak value (**b**). CF biaxial non-crimp fabric ± 45°, ST 0°, nine samples.

The geometry data of the time-dependent shear deformation can be imported into a software environment for statistical calculations and graphical output. The software used in this application case was “R” [41]. As described in Section 3.1, all data of a test cycle are leveled based on a first time step (initial state of the shear test), in order to eliminate the intrinsic error of the measurement system. The cleansed data can be used in a further process to visualize the deflection of the laser beam over time as a 3D area. Figure 16 shows the characteristics of the folds including their minima and maxima over a constant sample width. A color gradation allows the assignment of numerical values to different fold heights. Moreover, the amplitude maxima and minima of each time step can be illustrated both in a separate diagram (Figure 17a) and as a sum of their amount (i.e., as peak-to-peak value, Figure 17b) as a function of time and the shear angle.

**Figure 18:**
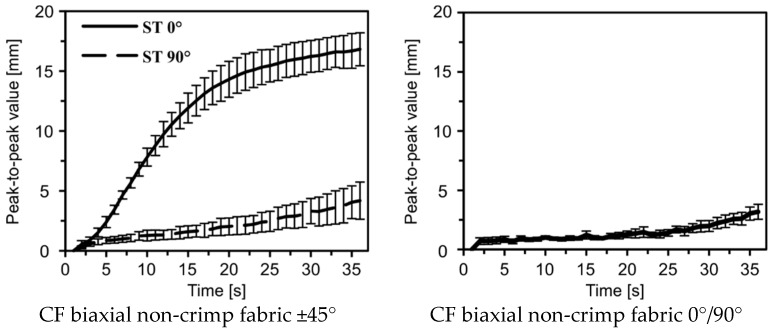

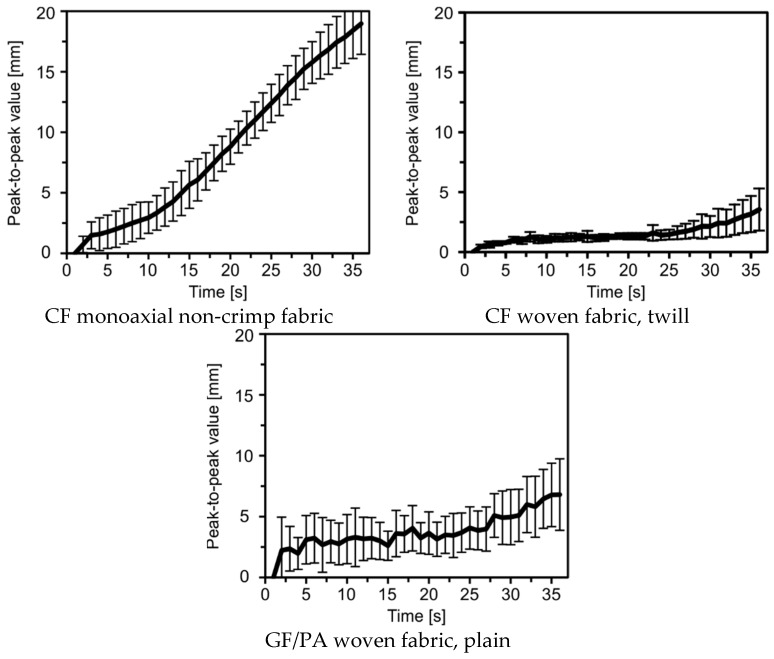
Averaged peak-to-peak value with standard deviations of each material over time.

The amplitudes of maxima and minima revealed qualitatively different curve progressions. As an example, a maximum is initially formed in the case of sample 6, which decreased after about 12 s, as the minimum increased from this point forward. In contrast, the amplitude progression of sample 4 shows a continuous maximum, whereas the minimum remained relatively low. The analysis of the amplitude curve explains the differences of samples of a material variant in shear behavior (cf. Figure 15). The evaluation of the peak-to-peak value, however, clearly indicates qualitatively comparable curve progressions and thus the reproducibility of the developed shear deformation. The deviations that occur (Figure 17b) result from inaccuracies in sample preparation as described above and cannot be avoided even if the test is performed with great accuracy. Figure 18 shows the averaged results of the peak-to-peak values and the standard deviations over time for each material and material direction. Besides the absolute measured heights, which strongly differ, a closer view on reproducibility is possible. It is obvious that, for the CF-based woven and biaxial non-crimp fabrics, the standard deviations at the beginning of picture frame testing were lower than at higher deflections. Furthermore, the increase in standard deviation seems to be correlated with the beginning of folding. For the CF-based monoaxial non-crimp fabric and the GF/PA-based woven fabric, the standard deviations were nearly constant over time, but significantly higher than for the other materials. Especially for the GF/PA-based woven fabric, reflections occurred during testing, influencing the base data set and leading to the obtained high standard deviations. Furthermore, the observed results for CF monoaxial non-crimp fabric need to be considered critically as earlier investigations [26] revealed that shearing is not an intrinsic behavior of monoaxial non-crimp fabrics. Additionally, sample preparation is much more challenging, given by the loose construction. Overall, the high standard deviations are caused by material-specific behavior, not by the accuracy of the extended picture frame test. Hence, the additional optical evaluation of fold height is more reliable than an objective evaluation of folding.

This provides the user with a tool for automatically detecting the exact point of time of fold formation in a reproducible manner and for relating it to a critical shear angle. Depending on the geometry of the construction part to be produced and on the specific requirements, a limit value can be defined for a critical fold height. If this limit value is exceeded, the critical shear angle can be assigned, based on the time step. Consequently, the shear angle serves as a reliable decision criterion in kinematic draping simulations.

## 4. Conclusions

One of the most important requirements for the application of fiber-reinforced composites in large-scale productions is the reproducible production of FRP components with a constant quality. Reducing the time and resources necessary for the development of prototypes and to enhance virtual component development, appropriate methods for material tests and forming simulations are required. These methods, in turn, demand an application-oriented and, even more importantly, conclusive test technology to determine the characteristic material properties.

For kinematic draping simulations, a critical shear angle needs to be determined. Practical experiments have revealed that simulation parameters cannot be clearly defined for all material constructions based on the shear force/shear angle curve of the picture frame test. Instead, additional information is necessary to comprehensively characterize shear behavior.

The hardware and software developed for the recording and graphical evaluation of shear deformations by means of a laser beam enables fold formations to be automatically detected and quantified, and to be related to shear force/shear angle curves. Hence, a tool offering a wide variety of additional information for analyzing the shear behavior of reinforcing textiles is available. In the future, this tool may help users in practice to decide on suitable materials and to improve transparency in the design and construction process.

The extended test setup can be integrated into the currently established picture frame test without significant efforts. Moreover, no additional sample preparation is required, which results in considerable time savings as compared to currently available 3D deformation measuring systems. Therefore, this method is a cost-efficient solution tailored to small and medium-sized companies. Moreover, the accuracy of the extended picture frame test stand has been demonstrated for several high performance textile fabrics. Additionally, further knowledge was gained regarding the influence of fabric construction, e.g., the linear density of sewing thread, on deformation behavior under shear stress. This is a very interesting inspiration for more detailed investigations in future, because this affects not only the users of such materials, but also the producers. Another interesting application is the possibility to evaluate FE-based simulation by comparison of the attained surface deformations.

## Figures and Tables

**Table 1:** Material specifications.

Identification	Appearance	Mass Per Unit Area	Warp Yarn	Weft Yarn	Sewing Thread		
					Material/Orientation	Linear Density	Stitch Length
		(g/m²)	(tex)	(tex)	(°)	(dtex)	(mm)
CF biaxial non-crimp fabric ±45°	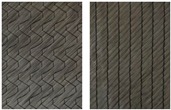	300	1600	1600	PES/0	36	2.9
CF biaxial non-crimp fabric 0°/90°	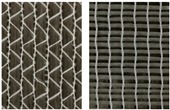	349	800	400	PES/0	180	2.5
CF monoaxial non-crimp fabric 0°	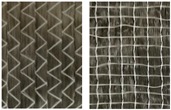	330	1600	-	PES/0 PES/90	820 340	2.5
CF woven fabric, twill 2/2	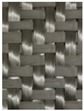	400	800	800	-	-	-
GF/PA woven fabric, plain	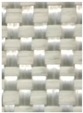	625	1800	1800	-	-	-

^1^ For non-crimp fabrics the front and back and for woven fabrics, only the fronts are shown

**Table 2:** Evaluation of reference geometry within the extended picture frame test stand.

Criteria	Peak 1	Peak 2	Peak 3	Peak 4	Peak 5
Nominal value (mm)	10.00	5.00	20.00	15.00	10.00
Measured value (mm)	9.59	4.83	19.72	14.21	10.23
Absolute Deviation (%)	4.12	3.44	1.42	5.28	2.35
